# Isolation and characterization of pectinase-producing bacteria (*Serratia marcescens)* from avocado peel waste for juice clarification

**DOI:** 10.1186/s12866-022-02536-8

**Published:** 2022-05-24

**Authors:** Setegn Haile, Chandran Masi, Mesfin Tafesse

**Affiliations:** 1 Department of Biotechnology, College of Biological and Chemical Engineering, Addis Ababa Science and Technology University, P.O. Box 16417, Addis Ababa, Ethiopia; 2 Center of Excellence for Biotechnology and Bioprocess, Addis Ababa Science and Technology University, P.O. Box 16417, Addis Ababa, Ethiopia

**Keywords:** Bacteria, Clarification, Polysaccharide, Pectinase activity, *Serratia marcescens*

## Abstract

**Background:**

Bacterial pectinase is an enzyme that could be employed in numerous sectors to break down pectin polysaccharide compounds. The goal of this study is to find pectinase-producing bacteria in avocado peel waste and see if the pectinase enzyme produced can be used to make fruit juice clarification.

**Results:**

The researchers isolated four different bacterial strains from avocado peel waste samples. The potential two bacterial isolates that were identified as being *Serratia marcescens* and *Lysinibacillus macrolides.* Finally, the analysis of pectinase production and its application in fruit juice clarification were performed using one of the bacterial strains of *Serratia marcescens*. The clear apple, lemon, and mango juices were further processed to assess each juice's properties. The highest antioxidant activity was recorded in lemon juice samples. The lemon juice showed the highest total titratable acidity and total phenol content. Apple juices contained the highest total soluble solids, reducing sugar content, and viscosity and the mango juices have the maximum pH value recorded.

**Conclusions:**

The pectinase isolated from the bacterium *Serratia marcescens* could clear fruit juices. This pectinase needs to be studied more to make sure it works better in the fruit industry and other businesses.

## Introduction

Enzymes are biological catalysts that help chemical reactions occur under various physicochemical conditions. All enzymes are protein in nature, but each has a unique performance function [1–3]. Enzymes were first identified in the mid-nineteenth century and the first to recognize the technical potential of cultivated enzymes and commercialize primarily using fungal enzymes, but 20 years later, Boidin and Affront in France pioneered the synthesis of bacterial enzymes [4, 5].

Today, industrial enzyme technology relies on microbial sources such as bacteria and yeasts. These microorganisms are essential in the production of pectinase enzymes which find applications in biotechnological processes that use pectin as a carbon source [6, 7]. Pectin is a component of the plant's cell wall and middle lamella, and a very thin extracellular layer that connects the young cells [8, 9].

The pectin substances are complex colloidal acid polysaccharides with a long galacturonic acid pillar chain and glycoside bonds. Seven polysaccharides and 17 monosaccharides, such as d-Glucuronic acid, l-Fucose, d-Glucose, d-Mannose, and d-Xylose, are present in these chains of pectin compounds [6, 10, 11]. Pectic acid, pectinic acid, pectin, and protopectin are the four types of pectin substances used as substrates in pectinase processing [12, 13]. The solubility of pectic substances in water was one of the most relevant criteria used to identify those [14, 15].

Only 25% of the Microbial pectinase enzyme was used in the food and industrial sectors around the world, even though the market kept growing [16, 17]. Pectinase finds applications in the extraction of fruit juice, clarification of juice, refining of vegetable fibers, degumming of natural fibers, and wastewater treatment [18, 19]. It also speeds up tea fermentation and eliminates the foam-forming property of instant tea powder by destroying the pectin present in tea powder. Even though they aren't just used to make coffee, they're also used to remove the mucilaginous layer from coffee beans [15, 20].

Fungal organisms produce the vast majority of pectinase used in the industrial environment. The enzyme is used mostly for the degradation of pectic compounds in a variety of industrial sectors because of which their demand has increased in recent years [11, 21]. To address this demand for pectinase, a bacterial source of pectinase could be used. Pectinases break down pectin, causing a decrease in viscosity and the formation of clusters, making centrifugation or filtration easier. As a result, the juice has a more transparent appearance and a more intense taste and color [22, 23]. The efficiency of Pectinolytic enzymes in fruit juice clarity is, however, reliant on the amount of pectin and pectinases available in the substrate, resulting in better juice extraction and clarification [14, 24]. The major sources of microorganisms used to produce pectinase are pectin-containing fruits and their peels, such as orange pulp, avocado peel, potatoes, tomatoes, sugar beet pulp, peaches, strawberries, lemon carrots, and banana peels. A bacterium has not been found in avocados, but pectic and pectin compounds have been found in a lot of research on avocados [7, 12].

**Fig. 1:**
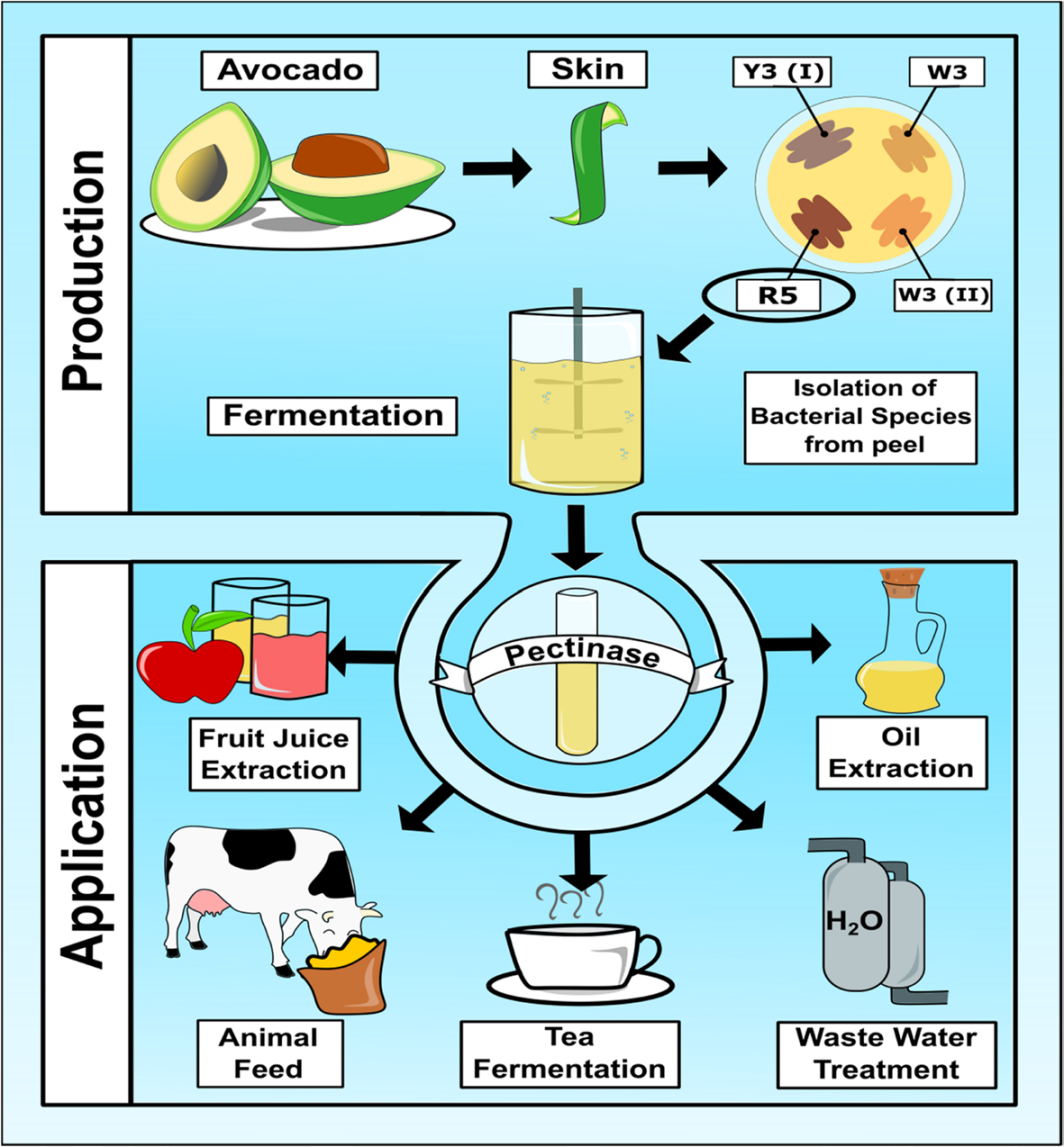
Isolation of pectinase bacteria, production, and application of pectinase

Pectinases are used in acidic and alkaline environments and are especially useful in the food and textile industries [25, 26]. Pectinases and their uses are being studied in global research sectors to get optimal fastened activity with enzymes. Pectinase has a wide range of applications, which has increased global demand. The uses of these enzymes are depicted in Fig. 1. There is a lot of value in the pectinase enzyme because it makes fruit juice clear. The main goals of this research were to find and study pectinase-producing bacteria from avocado peel wastes (*Serratia marcescens*), and improve the clarity of the fruit juice.

## Results

### Isolation of bacteria from avocado peel

**Fig. 2:**
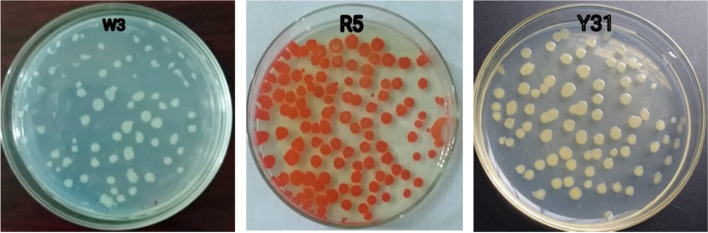
Single Colony isolation by Pour Plate Technique using pectin agar plate

It was used to isolate bacteria from avocado peel waste using serial dilution, pour plating, and streak isolation methods. They were sub-cultured into a new growth medium to obtain a pure isolate. The four pure isolates were obtained after extensive isolation techniques. The pure bacterial isolates were labeled as white colony (W3, W32) red colony (R5), and yellow colony Y31 to make it easier to distinguish between them [Fig. 2].

### Primary and secondary screening

**Fig. 3:**
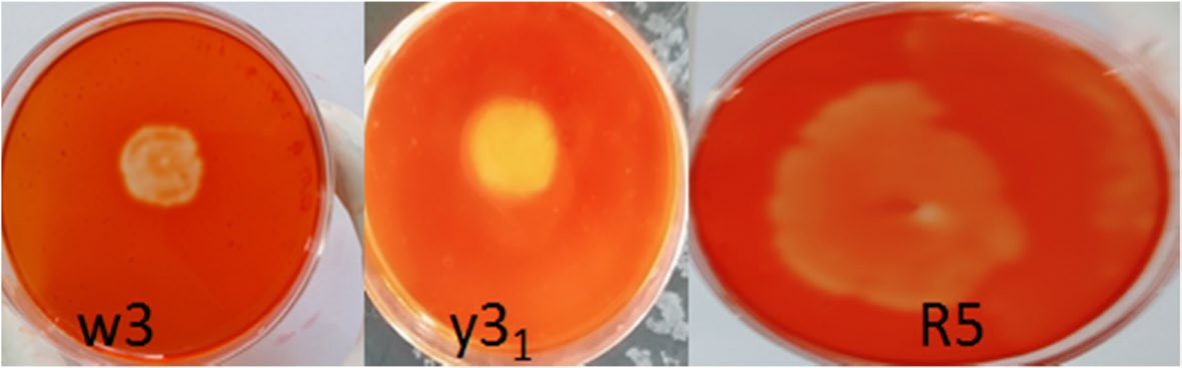
Primary screening of Pectinase producing bacteria isolates using pectin agar plate

There was a strong (hydrolysis) zone among all four bacterial isolates, indicating the existence of pectinase activities. The diameter of each hydrolysis zone was calculated to determine the potential bacterial isolate [Fig. 3]. Isolate R5 measured the largest diameter around the colony at 20.54 ± 1.32 mm [Table 1]. The activities of crude pectinase were measured in the secondary screening. The selected isolates from the primary screening method were subjected to fermentation in a suitable medium, and their behaviors were assessed to be further screened. A liquid sample (0.4 ml) was taken to assess pectinase activation by using phosphate buffer after 24 h of incubation in the production media. Isolate R5 had the highest pectinase activity of 5.410 ± 14 mol/ml/min, isolate W3 had the second highest at 3.090 ± 17 mol/ml/min, and isolate Y31 had the lowest at 2.490 ± 23 mol/ml/min [Table 2].

**Table 1:** Primary screening of pectinase producing bacterial isolates using zone of inhibition methods

*S. No*	*Isolate code*	*Clear Zone Diameter (in mm)*
1	W3	14.08 ± 0.75
2	W32	10.24 ± 0.54
3	Y31	12.86 ± 1.80
4	R5	20.54 ± 1.32

^*^SD represented standard deviation

**Table 2:** Secondary screening of crude Pectinase activities

*S. No*	*Isolate code*	*Pectinase activities (U/ml)*	*Relative activities (%)*
1	W3	3.09 ± 0.17	57.12
2	Y31	2.49 ± 0.23	46.02
3	R5	5.41 ± 0.14	100

Values is mean ± standard deviation of replicates

### Morphological and biochemical identification of bacterial isolates

Using Bergey's Manual of Determinative Bacteriology [27] isolates R5 and Y31 were discovered to be Gram-negative bacteria, whereas isolate W3 was confirmed to be Gram-positive bacteria. The isolates were tentatively described as *Bacillus species, Serratia species,* and *Erwinia* species [Table 3] using ABIS-online software. We chose gram-positive bacteria from W3 and gram-negative bacteria from R5 for further research.

**Table 3:** Morphological and biochemical characterization of bacterial isolates

*Characteristic*	*Bacterial isolates*		
	***R5***	***W3***	***Y31***
**Morphological features**			
Color	Creamy red	White	Yellow
Shape	Rod	Rod	Long rod
Surface	Smooth	Smooth	Smooth
Opacity	Opaque	Opaque	Opaque
Gram staining	Negative	Positive	Negative
**Biochemical characteristic**			
Sucrose hydrolysis	Positive	Positive	Positive
Lactose hydrolysis	Negative	Negative	Positive
Glucose hydrolysis	Positive	Positive	Positive
Indole test	Negative	Negative	Negative
Methyl red test	Negative	Positive	Positive
Urease test	Negative	Negative	Positive
Voges—Proskauer test	Positive	Negative	Positive
Casein hydrolysis	Negative	Positive	Negative
Citrate utilization test	Positive	Negative	Negative
H_2_S test	Negative	Negative	Positive
Catalase test	Positive	Positive	Positive
Similarity of bacteria	*Serratia spp*	*Bacillus spp*	*Erwinia spp*

### Molecular identification of isolates

**Fig. 4:**
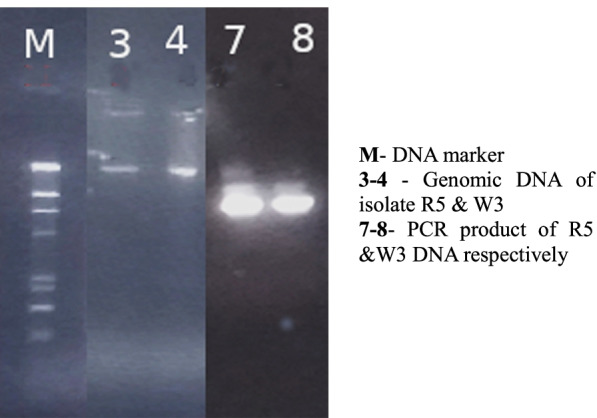
A gel documentation system was used to acquire an image of an agarose gel dyed with ethidium bromide - DNA marker (M), Genomic DNA extracted - 3(R5) and 4 (W3), and PCR amplification product of bacterial genomic DNA - 7 (R5) and 8 (W3)

**Fig. 5:**
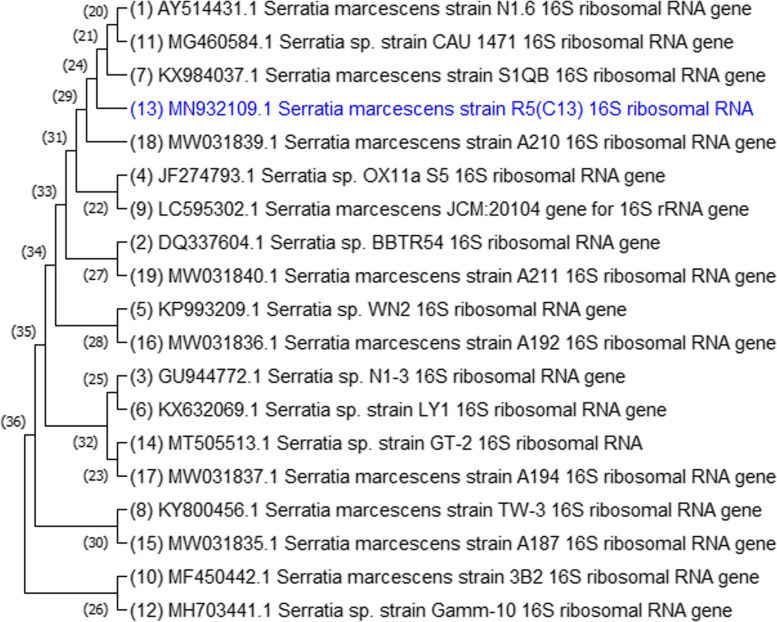
Phylogenetic tree constructed based on 16S rRNA gene sequences of Serratia marcescens _R5 with other Serratia species obtained from GenBank database

**Fig. 6:**
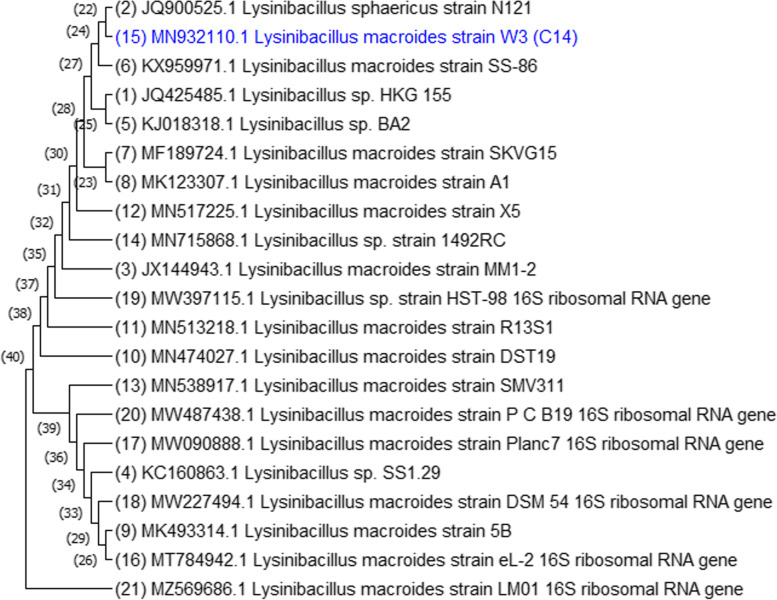
Phylogenetic tree constructed based on 16S rRNA gene sequences of *Lysinibacillus macroides*_W3 with other *Lysinibacillus species* obtained from Gene Bank database

The 16S rRNA gene was amplified by polymerase chain reaction (PCR) using genomic DNA from selected bacterial isolates (R5 and W3) as templates. The genomic DNA and PCR amplification products were analyzed using agarose gel electrophoresis compared with DNA marker isolate R5, and W3, as shown in Fig. 4. To obtain the right sequences, the PCR products were filtered and sequenced. Each isolate's sequences were uploaded to the NCBI database and compared to previously published sequences. The closest neighbors of the isolates R5 (MN932109.1) *Serratia marcescens* and W3 (MN932110.1) *Lysinibacillus macrolides* were queried using NCBI BLAST (HTTP:// www.ncbi.nlm.nih.gov/Blast). The nucleotide *Serratia marcescens* strain 16S ribosomal RNA gene sequences from R5 quest (mBLAST, NCBI) showed 99% homology (Fig. 5). The sequences of the W3 search (mBLAST, NCBI) showed 86% similarity to the nucleotide of *Lysinibacillus sp*., and 85% similarity to the nucleotide of *Lysinibacillus macrolides* in the phylogenetic tree (Fig. 6).

### Production of pectinase and its optimization

The development of a broad hydrolysis zone on pectin agar plates was used to characterize the screened Pectinolytic bacterial isolates. Fermentation and quantitative screening of isolate R5-*Serratia marcescens* were performed, measured by pectinase activity. After 48 h of fermentation, the maximum pectinase activity was 5.41 ± 0.14 µmol/ml/min [Table 2].

### Effect of fermentation time on pectinase production

To get the most pectinase production from isolate R5-*Serratia marcescens*, we selected five incubation times (hours) that were used. Pectinase activity increased gradually over 24 h of incubation until optimum pectinase activity was achieved. After the optimum incubation period, the pectinase activities began to decrease. In this analysis, the optimum fermentation period for pectinase development was found to be 72 h, and the highest pectinase activities were found to be 6.86 ± 0.32 µmol/ml/min of released glucose [Table 4] when pectin was used as a substrate.

**Table 4:** Effect of incubation time on pectinase activities in isolateR5 (*Serratia marcescens)*

*Incubation time(hours)*	*Pectinase activities ( U/ml)*	*Relative Activities (%)*
24	5.21 ± 0.54	75.95
48	5.54 ± 0.48	80.76
72	6.86 ± 0.32	100
96	4.83 ± 0.78	70.41
120	4.54 ± 0.92	66.18

### Effect of pH on pectinase production

The effect of different pH of fermentation medium on pectinase production by bacterial isolate R5-*Serratia marcescens* was studied. The pectinase production could be affected due to the variations in pH medium. The pectinase activity increased when we reached the optimum pH and decreased after the optimum pH value. The production medium, which was adjusted to pH 8 produced maximum pectinase activity (8.84 ± 0.34 µmol/ml/min), followed by pH 7 (6.66 ± 0.73 µmol/ml/min) [Table 5].

**Table 5:** Effect of pH on pectinase Activities in isolate R5 (*Serratia marcescens)*

pH	*Pectinase activities ( U/ml)*	*Relative Activities (%)*
5	1.43 ± 0.42	16.18
6	3.84 ± 0.54	43.43
7	6.66 ± 0.73	75.34
8	8.84 ± 0.34	100
9	6.34 ± 0.92	71.72
10	3.42 ± 0.64	38.69

### Effect of temperature on pectinase production

The effects of temperature on pectinase production by bacterial isolate R5-*Serratia marcescens* indicated that maximum pectinase activity was obtained at 35 °C. As a result, pectinase activity increased as temperature increased and slightly decreased after crossing the optimal temperature of 35 °C. However, pectinase activity was not completely lost even if the temperature increased to 50 °C. Although the maximum pectinase activity was 7.76 ± 56 µmol/ml/min of glucose released at 35 °C, pectinase activities above the optimal temperature were frequently higher than those below the optimal temperature [Table 6].

**Table 6:** Effect of Temperature on pectinase activities in isolate R5 (*Serratia marcescens)*

**Temperature(**^**o**^C)	***Pectinase activities ( U/ml)***	***Relative Activities (%)***
25	5.24 ± 0.98	67.53
30	6.56 ± 0.34	84.54
35	7.76 ± 0.56	100
40	6.93 ± 0.32	89.30
45	5.89 ± 0.34	75.90
50	5.42 ± 0.12	69.84

### Effect of substrate concentration on pectinase production

The effect of substrate concentration on pectinase production by bacterial isolate R5-*Serratia marcescens* showed antagonistic effects after 1% of pectin (8.91 ± 0.23 µmol/ml/min). As a result, observed in Table 7, pectinase activity increased with increased substrate concentration (pectin) up to the optimal concentration and decreased after the optimal substrate concentration.

**Table 7:** Effect of substrate concentration on pectinase production in isolate R5 (*Serratia marcescens)*

*Substrate Concentration (%)*	*Pectinase activities ( U/ml)*	*Relative Activities (%)*
0.25	4.99 ± 0.30	56.00
0.5	7.53 ± 0.30	84.51
0.75	7.92 ± 0.31	88.66
1	8.91 ± 0.23	100
1.25	8.22 ± 0.46	92.56
1.5	7.94 ± 0.80	88.72

### Purification of pectinase and determining protein concentration

Pectinase precipitation is the preferred concentration method and an ideal step in the purification process. One of the most well-known and widely used methods of purifying and concentrating pectinase, especially at the laboratory scale, is salting-out proteins, particularly ammonium sulfate. Pectinase was isolated from isolated R5-*Serratia marcescens* and subjected to various saturation levels of ammonium sulfate varying from 30–90%. The pectinase activities and protein content partially purified by ammonium sulfate are presented in Table 8.

**Table 8:** Purification profile of pectinase production in isolate R5 (*Serratia marcescens)*

*Purification*	*Total Volume (ml)*	Total activity* (U/ml)	*Total Protein*^*#*^ *(mg)*	*Specific activity*^*@*^ *(U/mg)*
Crude pectinase	200	1935	57.45	33.68
Ammonium Sulphate (70%)	75	773.85	20.1	38.5
Dialysis	40	473.28	10	47.32

^*^Total activity = enzymes activity x total volume

^#^Total protein = protein x total volume

^@^Specific activity = total enzyme activity/ total protein content

### Application of pectinases in fruit juice clarification and yield

This study tried to determine the effect of pectinase on the volume of juice, juice yield, and juice clarity in terms of transmittance by using apple, lemon, and mango fruits. The experiments were carried out with crude pectinase, partially purified, and water as control. According to the results shown in Table 9, the volume of lemon juice was enhanced by three folds from control to crude pectinase (10.0 -13.0 ml) and two folds increment from purified pectinase (13.0 -14.5 ml), and 86.67% of yield. Using mango juice, the same experiment was carried out. The amount of mango juice increased twofold from control to crude pectinase enzyme (8.0–10.0 ml) after 1 h of pectinase and water treatment and increased almost threefold from crude pectinase to purified pectinase (10.0 -13.5 ml), and 66.67% of yield.

**Table 9:** Application of pectinase Vs juice yield

*Fruits (15 ml)*	*Characteristics*	*Crude Pectinase*	*Purified Pectinase*	*Control*
Apple	Volume (ml)	11	14	8
	Yield %	73.33%	93.33%	53.33%
Lemon	Volume (ml)	13	14.5	10
	Yield %	86.67%	96.67%	66.67%
Mango	Volume (ml)	10	13.5	8
	Yield %	66.67%	90%	53.33%

The same experiment was performed to express the yield of juice in terms of percentages. As the above table indicates, the rate of juice yield was improved, starting from control to purified pectinase (Table 9). The yield variation of apple juice between control and crude pectinase was 20%. Similarly, the 20% of apple juice yield variation was recorded between crude pectinase and purified pectinase. Therefore, apple fruit showed consistent variation from control to crude and crude to purified pectinase. The yield of lemon juice variation between control and crude pectinase was 20%, but the divergence between crude pectinase and purified pectinase was recorded as 10%. As a result, there were no consistent differences in lemon juice yield from control to pure pectinase. In mango juice, there was a 13.34% difference in yield from control to crude pectinase and a 23.33% difference in juice output between crude pectinase and purified pectinase.

### Effect of pectinase on juice clarification

**Fig. 7:**
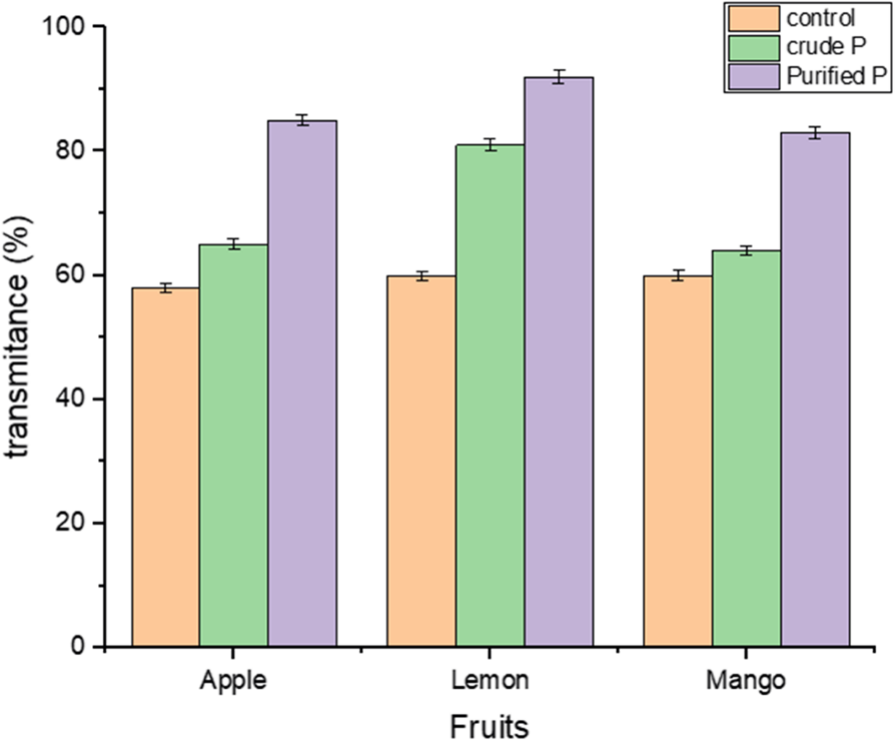
Effect of pectinase on juice clarity in terms of transmittances

**Fig. 8:**
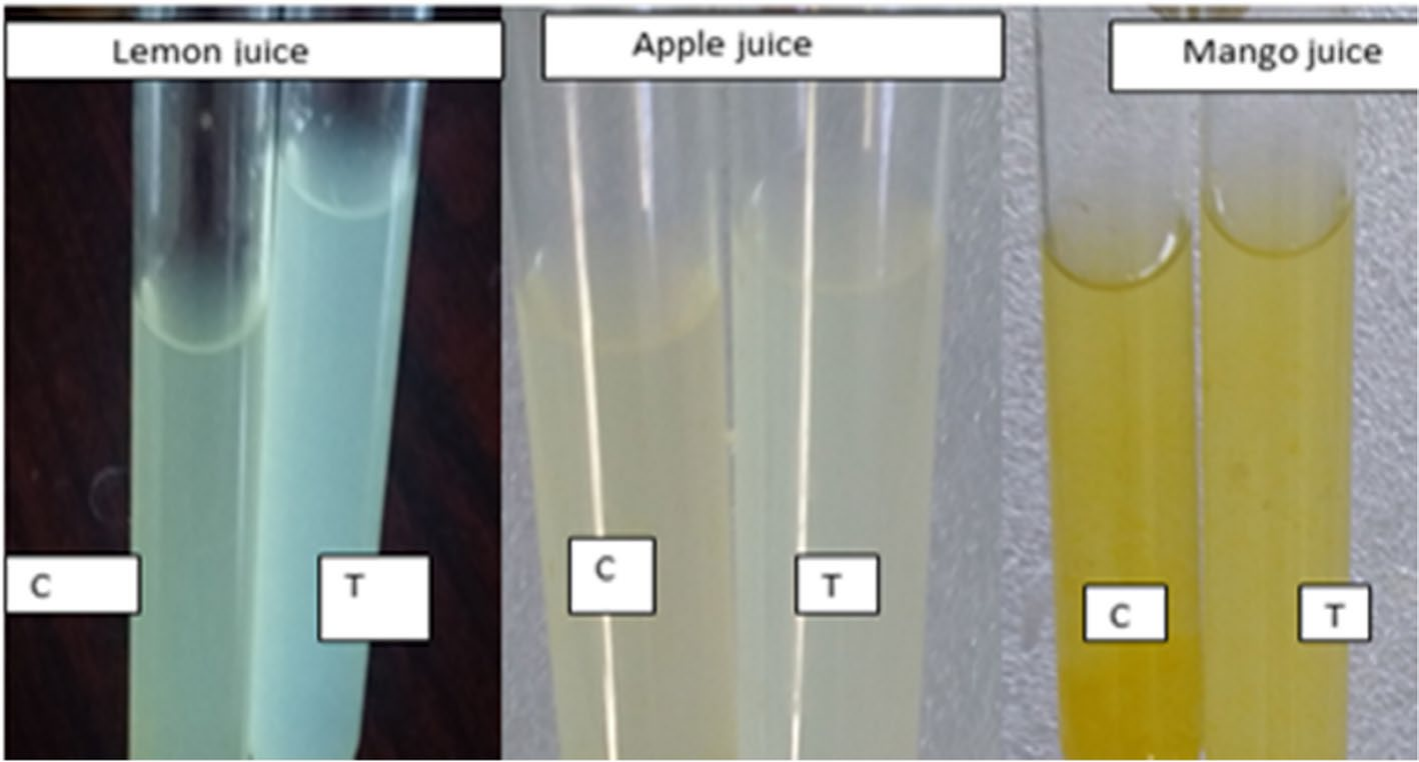
The effect of pectinase on fruit juice clarification: Contol – C (Before), Test – T (After clarification)

The transmittances of the clarified juice determined the effect of pectinase on juice clarity in terms of percentage (Figs. 7 and 8). As shown three different fruits [apple, lemon, and mango] were subjected to crude and purified pectinase by taking water as control, the maximum clarity of juice was obtained was 95.82% of transmittance for lemon fruit. When compared to the result achieved with crude pectinase, which was 78.99% of juice clarity transmission, the transmittance of lemon juice treated with purified pectinase was 16.83% higher.

### Untreated and treated juice properties

The viscosity, pH and acidity, total soluble solids (TSS), total titratable acidity (TTA), reducing sugar content, pectin presence test, Total phenolic content (TPC), and Antioxidant activity of three different fruits [apple, lemon, and mango juice] were evaluated, and the results were compared to the raw sample (untreated juice is a control), as shown in Tables 10 and 11. The pH of each fruit juice sample decreased with pectinase, and the total titrable acidity (TTA) increased after the juice was clarified by pectinase, as shown in Table 10. These results were probably related to pectin degradation and liberation of galacturonic acids after pectinase treatment. The reducing sugar content of each fruit juice sample increased after treatment by pectinase (Table 10), which is probably related to the liberation of reducing sugar after the degradation of pectin compounds. Because pectinase degrades polysaccharides (pectin), the viscosity of each fruit juice sample was dramatically reduced after treatment with pectinase, as shown in Table 10. It reduced the presence of cohesive network structure in fruit juice samples. The result of the total phenol content of each fruit juice sample also showed a decreasing trend after the treatment of the juice with pectinase, as shown in Table 11. The hydrolysis of pectin led to the decomposition of phenolic compounds and decreased their content in fluids.

**Table 10:** Total Titratable Acidity (TTA), Total Soluble Solids (TSS), Reducing Sugar content (RSC), pH, Viscosity, and Pectin presence test of the untreated and treated fruit juice samples

*Parameters*	*TTA*	*TSS*	*RSC*	*pH*	*Viscosity*	*Pectin test*
UnLMJ	1.30 ± 0.02	11.2 ± 0.05	45.34 ± 0.01	3.9 ± 0.21	1.80 ± 0.04	Have
LMJ	1.85 ± 0.10	10.0 ± 0.01	48.02 ± 0.03	3.2 ± 0.30	1.23 ± 0.10	None
UnMANJ	1.40 ± 0.03	15.33 ± 0.06	55.04 ± 0.01	5.8 ± 0.03	1.50 ± 0.20	Have
MANJ	1.60 ± 0.01	12.24 ± 0.01	57.21 ± 0.03	4.9 ± 0.01	1.02 ± 0.30	None
UnAPPJ	1.09 ± 0.2	17.22 ± 0.08	59.32 ± 0.02	4.2 ± 0.11	1.71 ± 0.04	Have
APPJ	1.34 ± 0.03	15.43 ± 0.04	62.05 ± 0.01	3.8 ± 0.20	1.54 ± 0.06	None

*UnLMJ* Untreated lemon juice (control), *LMJ* Treated lemon juice, *UnMANJ* Untreated mango juice, *MANJ* Treated mango juice, *UnAPPJ* Untreated apple juice, *APPJ* Treated apple juice

**Table 11:** Total phenolic content (TPC), Antioxidant activity (*ABTS-RSA& DPPH – RSA)* Untreated and treated fruit juice samples

*Parameters*	*Total phenol content*	*Antioxidant activity*	
		***ABTS-RSA***	***DPPH – RSA***
UnLMJ	2.55 ± 0.01	91 ± 0.30	94 ± 0.41
LMJ	2.03 ± 0.04	88 ± 0.21	89 ± 0.12
UnMANJ	3.50 ± 0.09	78 ± 0.11	80 ± 0.31
MANJ	2.02 ± 0.05	70 ± 0.32	75 ± 0.11
UnAPPJ	1.99 ± 0.02	89 ± 0.43	89 ± 0.21
APPJ	1.05 ± 0.04	85 ± 0.31	84 ± 0.33

*UnLMJ* Untreated lemon juice (control), *LMJ* Treated lemon juice, *UnMANJ* Untreated mango juice, *MANJ* Treated mango juice, *UnAPPJ* Untreated apple juice, *APPJ* Treated apple juice

## Discussion

The hydrolysis of isolate R5 in primary screening is almost identical to that of previous studies [2, 28, 29], which obtained 22.8 mm and 20.5 mm, respectively. This may be due to the ability of bacteria to break down pectin. The hydrolysis zone of isolate (W3), in particular, was linked to the last tomato isolate [6, 23, 30]. Similarly, the results of each isolate's secondary screening method are more similar to the previous investigation of decomposing orange peels; this resulted in the sources of the samples mentioned by [22, 31]. Pectinase-producing bacteria were isolated from coffee pulp [32], and the results of the secondary screening method were lower than isolate R5.

Biologists have used a series of biochemical tests to distinguish closely related bacteria in the detection of bacteria [27]. Bergey's Manual of Determinative Bacteriology, based on their physical and biochemical properties, the ABIS-online software was used to detect Pectinolytic bacteria [27, 29]. In molecular identification, the neighbor-joining method was used to build the phylogenic tree based on the 16S rRNA gene sequences of isolates R5 and W3 and associated nucleotide sequences [2, 22, 33].

Different researchers investigated the molecular characterization of pectinase production with Serratia species. *Serratia rubidaea* (E9.HM585373) was isolated from tomato fruits and characterized using the 16S rRNA method. As reported by Abd-alla et al., [6] these bacterial species were found to be producing polygalacturonases at a temperature of 40 °C. *Serratia rubidaea* and *Serratia marcescens* are different from each other in species labels, but both are used to produce pectinase, even though their isolate sources are different. About 20 enzyme-producing bacterial strains were isolated from municipal solid waste using the 16S rRNA method. Among those bacterial strains, most of the strains were *Bacillus species*. Sarreen et al., [34] discovered that only two bacterial strains, *Serratia marcescens* (MH194203) and *Lysinibacillus species* (MH194187), were associated with this discovery. *Serratia oryzaestrain* S32 (SOZ00000000.1) was also found in lake water. This showed that bacterial strains could make pectinase, which was found by Huguevieux et al., [35].

The pectinase, which was produced in just 24 h of incubation, was two times more potent than the 2.43 U/ml pectinase obtained by *Bacillus sonorensis* in the same incubation period [11, 36, 37]. The enzyme activity results obtained after 72 h were similar to those obtained by Jayani et al., [38] who obtained 7.88 U/ml of pectinase activity after 72 h of fermentation time using *Bacillus sphaericus*. This study finding shows that this bacterial isolate, R5-*Serratia marcescens*, requires an alkaline condition in pectinase production processes [24, 39]. According to the investigations by Mohandas et al., [36] the highest pectinase activity, 2.43 U/ml, was recorded at alkaline pH 8 by *Bacillus sonorensis*. According to Sohail and Latif [40], the optimal poly galacturonase production of *Bacillus mojavensis* was at pH 8.0*. Streptomyces* species require a slightly alkaline condition of pH 8.5 for maximum pectinase production.

Temperature is one of the essential parameters essential for the success of pectinase production. According to Kothari and Baig [41], the maximum polygalacturonase activities were produced at a temperature of 35 °C by *Erwiniacarotovora*. In the same bacterial species (*Erwinia* spp*)*, the highest pectinase (polygalacturonase and pectin lysate) activity was recorded at a temperature of 35 °C [5, 42]. Other recent investigations indicated that the highest amount of pectinase produced by *Entero bactertabaci* NR1466677 was at the optimal temperature of 35 °C [9, 30].

In this study, various concentrations of pectin substrate were used as a carbon source for bacterial growth and were subjected to a fermentation medium to produce pectinase. The pectinase activity data in this study appear to be comparable to the work of Darah et al., [21]. They obtained maximum polygalacturonase activity at 1% of the pectin concentration for *Entero bacteraerogenes*. Some bacterial species, such as *Entero bactertabaci* NR14667*,* could produce the highest pectinase activity at 0.3% of pectin concentration, as reported by Obafemi et al., [30]. More recently, maximum pectinase activity at 2% of citrus pectin concentration was also recorded for *Chryseo bacterium indologenes strain SD *[21, 43].

The unique activities of pectinase increased from crude to dialyzed pectinase. With 10 mg of protein concentration, the maximal activity was 47.32 U/mg, suggesting that the protein molecules separated by ammonium mainly contained the enzyme pectinase and that the proportion of protein other than pectinase was higher in the crude form of the enzyme [11, 31, 44]. Purification steps also resulted in the removal of interfering materials found in the crude cell-free sample, allowing for increased enzyme activity [13, 45]. From the application of pectinase, the volume of juice treated with both crude and purified pectinase varied with the type of fruit used in the process. As the results indicate, the highest juice volume was obtained from pectinase-treated lemon fruit, which might be due to the presence of solubility of pectin in lemon fruit [46, 47].

Similarly, the volume of juice variation from fruit to fruit that was treated with pectinase was investigated by using different fruits such as strawberry juice (7.0 – 10.0 ml), grape juice (15.0 -21.5 ml), apple juice (12.0—18.5 ml), peach apple juice (12.0—10.5 ml), chary apple juice (8.0 – 10.0 ml) and orange juice (9.0 – 10.0 ml) [29, 48]. The result of juice clarity treated with crude and purified pectinase was 1.99% and 18.82% more excellent than the previous report by Maktouf et al., [49], which was 77% of transmittance for pectinase using lemon fruit as the raw material for juice clarification. The result of mango juice transmittance obtained in this current experiment affirms the report made by Kumar et al., [50], who received 92.5% of transmittance after 150 min of incubation time. The effect of pectinase on juice clarification was also studied in apple juice. According to Yuan et al., [51], the clarity of apple juice treated with pectinase increased by 71.8% of transmittance. In their experiment, the clarity of apple juice rose to 84% transmittance.

Table 10 indicates that the total soluble solids decreased in each fruit juice sample after clarification. This can be due to the disintegration of solid compounds after the destruction network formed by pectinase [51, 52]. This was similar to the report of de Oliveira et al., [14], for apple juice clarification by pectinase. Table 10 shows that pectin was present in all untreated fruit juice samples, but pectin was not found in treated fruit juice samples by pectinase. A similar finding was reported by Hosseini et al., [53], for pomegranate juice clarification by free pectinase.

This result was similar to that of [37, 52], which was done on the clarification of apple juice by pectinase. In determining the antioxidant activity of each fruit juice sample, ABTS radical scavenging activities (ABTS-RSA) and DPPH radical scavenging activities (DPPH-RSA) of each fruit juice were evaluated, and the results were compared with the raw sample. As shown in Table 11, pectinase activities can change the phenolic compound profiles and the decomposition of another antioxidant by pectin hydrolysis [35, 52, 53].

## Conclusion

The fruits contain a high amount of pectin; the extraction of fruit juice has historically resulted in a cloudy, unappealing color and high viscosity. Researchers studied avocado wastes that were found to contain four distinct bacterial strains. One strain was classified as a *Serratia marcescens* based on morphological and biochemical characteristics. It's important to do more research on this enzyme to make sure and improve the efficiency of the bacteria for use in the fruit industry.

### Materials and methods

The major laboratory instruments used in this study were gel documentation, balance, centrifuge, Spectrophotometer, autoclave, refrigerator, microscope, water bath, water bath with shaker, and incubator.

### Sample collection

The avocado peel wastes were collected from the juice processing site of ECOPIA PLC, a private limited company, in Addis Ababa, Ethiopia. The samples were transferred into sterilized plastic bags and brought to the microbiology laboratory at AASTU. The sample containing bags were closed and stored in a 4℃ refrigerator until the analysis time [43].

### Serial dilutions

Homogenized one gram (1 g) of the avocado peel wastes sample was suspended in 9 ml of sterilized distilled water and was properly mixed. The mixture of 1 g avocado peel waste sample and 9 ml of distilled water were serially diluted from 10^–1^ to 10^–6^ in test tubes [54]. Serial dilution and spread plate methods were the techniques used to isolate the target to pectinase enzyme-producing bacteria using Nutrient agar media [55].

### Isolation and purification

The growth of bacterial colonies was observed after 24 h of incubation time. The next task was the purification and preservation of the culture. The individual colonies with similar character and size were isolated from culture plates and transferred to new agar plates to obtain pure colonies by the repeated streaking method. The purity of the test isolate was assessed using colony morphology and microscopy; pure colonies of bacteria were preserved with 20% of glycerol and stored at -80 °C for further study [56].

### Primary screening of pectinase producing bacteria isolates

All pure colonies from overnight cultured bacteria [freshly activated plates] were transferred to new pectin agar media and incubated at 30 °C for 48 h. At the end of incubation, 0.3% of Congo red solution was flooded onto the Petri dishes and left for 10 min. This solution formed a clear zone around the colonies, which indicates that bacterial isolates can produce pectinase and the diameter of clear zones is proportional to the bacteria’s relative pectinase production capacity [16].

### Secondary screening of pectinase producing bacteria isolates

The bacterial isolates showing maximum clear zone on primary screening media were considered the highest pectinase producer [54]. Those bacterial isolates with a higher clear area were subjected to submerged fermentation for pectinase production using the same medium as primary screening but without agar. The freshly cultured (stationary phase bacterial) isolates 0.2 ml in yeast extract broth were inoculated on 100 ml of sterilized production media (the media was fixed for 15 min at 121 °C) in 250 ml of the flask and incubated at 30 °C on a rotary shaker at 125 rpm for 48 h. At the end of incubation time, the media was transferred to a centrifuge tube and centrifuged at 10,000 rpm for 10 min. The supernatant was used as crude enzymes to evaluate the efficiency of bacterial isolates on the production of pectinase activities by using sodium acetate buffer pH 6.8 [1, 45].

### Determination of pectinase activity

Pectinase activity was determined by measuring the amount of released reducing sugar under assay conditions or by enzymatic degradation of pectin as described Nelson-Somogyi methods. The procedure was started by mixing 1 ml of substrate solution prepared by Phosphate buffer (pH 7) and 0.4 ml of specific supernatant enzyme in test tubes. Then, the mixed solution was incubated at 40 °C in the water bath for 40 min. After adding 0.3 ml of Somogyi copper reagent and mixture of the test tubes was set in a boiling water bath for 10 min. After incubation, the tubes were cooled to room temperature, and 0.3 ml of the Nelson arseno molybdate reagent was added. The solution was cooled to room temperature and measured at 540 nm after centrifuging at 10,000 rpm for 10 min by taking supernatant as the enzyme. The amount of released glucose per milliliter per minute was calculated using D-glucose (10–100 micromoles) from the standard curves. One unit of pectinase activities was defined as the amount of glucose released in the term of μmol of reducing sugar per ml per minute under Standard assay conditions [43, 57]. The pectinase activity was calculated using the following formula [58].

1 $$\mathrm{Pectinase\;Activities }\;(\mathrm{\mu mol}/\mathrm{ml}/\mathrm{min}) =\frac{\mathbf{R}\mathbf{G}\left({\varvec{\upmu}}\frac{\mathbf{m}\mathbf{o}\mathbf{l}}{\mathbf{m}\mathbf{l}}\right)\mathbf{*}\;\mathbf{T}\mathbf{V}\mathbf{A}(\mathbf{m}\mathbf{l})}{\mathbf{T}\left(\mathbf{m}\mathbf{i}\mathbf{n}\right)\mathbf{*}\;\mathbf{V}\mathbf{E}\mathbf{A}(\mathbf{m}\mathbf{l})}$$

where **RG** is released glucose obtained from D-glucose standard curve.

**TVA** is the total volume of assay.

**T** is the Incubation time.

**VEA** is the volume of enzyme used to assay.

### Morphological and biochemical tests for the identification of bacterial isolates

Single colonies grown on pectin agar media were smeared and examined under the microscope for morphological conformity using gram staining. The following biochemical tests were performed to identify bacterial isolates: carbohydrate fermentation tests, indole test, methyl red (MR) test, Vegas-Proskauer (VP) test, Citrate utilization test, hydrogen sulfide generation test, catalase test, and urease test [59].

### Molecular identification and PCR amplification of screened isolates

Molecular approaches were used to identify the prospective isolates that were examined and selected utilizing the primary and secondary screening procedures (R5 and W3). The genomic DNA of the isolates was extracted using the Bacterial Genomic DNA Extraction Kit, which was modified slightly from the manufacturer's procedure (QIAGEN, QIAamp DNA Mini Kit). The amplification process took 2:35 total time. On a 0.8% agarose gel dyed with a DNA-safe stain, the PCR products were seen. Finally, the PCR products were sequenced, and the obtained sequence data were analyzed using the basic local alignment search tool (BLAST) software (http://www.ncbi.nlm.nih.gov/blast) against the 16S ribosomal RNA sequence database, with the mega X software (http://www.ncbi.nlm.nih.gov/mahalik) to generate the phylogenetic tree from the national center for biotechnology [44].

### Production of crude pectinase by submerged fermentation

The pectinase production by submerged fermentation was conducted as described by Kumar A and Sharma R [50]. The bacterial isolate identified as a potential candidate was selected to produce this crude enzyme. About 2 ml of the three hours bacterial cultures (log phase bacteria) were inoculated to a pre-sterilized fermentation medium to maintain pH 8 [43] and incubated at 30 °C using a rotary shaker at 125 rpm for 48 h. At the closed fermentation time, the production medium was centrifuged at 10,000 rpm for 10 min. The clear supernatant was used for pectinases activities [47].

### Optimization of pectinase production

The production of pectinase was optimized by using four parameters namely, fermentation time, temperature, pH, and substrate concentration. The relative activity of each parameter was calculated as the percentage by using the following formula:-

2 $$\mathrm{Relative\;Activity}= \frac{{\varvec{A}}S}{MS}*100$$

where As = the activities of sample in µmol/ml

MS = the maximum activities of the sample in µmol/ml.

### Optimization of fermentation time on pectinase production

The production media was prepared at constant pH 7 and 1% substrate concentration to examine the influences of fermentation time on enzyme production by bacterial isolate. A Single colony of R5 isolate was inoculated in 15 ml of yeast extract pectin broth and incubated at a temperature of 30 °C overnight. The production media was sterilized at 121 °C for 15 min. This fixed production media was inoculated with 2 ml of overnight culture bacteria and incubated at a temperature of 30 °C using a rotary shaker with 125 rpm for 24 to 120 h. The activities were assayed in 24 h intervals [60].

### Optimization of pH on pectinase production

To investigate the influences of pH variation on enzyme production by bacterial isolate, the pH level of production media was adjusted from a pH 5 to pH 10 using 0.1 M sodium acetate and 0.1 M Sodium hydroxide [4] with a few modifications. The production media was sterilized at 121 °C for 15 min and inoculation with 2 ml of overnight bacterial cultured bacteria. This inoculated media was incubated at 30 °C using a rotary shaker with 125 rpm for 72 h, after which the enzyme activities were assayed.

### Optimization of temperature on pectinase production

Temperatures for enzyme production were maintained at the following temperatures to explore the effects of temperature change on enzyme production using bacterial isolate: 25, 30, 35, 40, 45, and 50 °C [61]. The production media (pH 8) was inoculated with 2 ml of overnight cultured bacteria and incubated using a rotary shaker at 125 rpm for 72 h. After the end of the fermentation times, the enzyme activities were assayed.

### Optimization of substrate concentration on pectinase production

To study the influences of substrate concentration on enzyme production using the bacterial isolate, the enzyme was produced by various concentrations of substrates (pectin). The concentrations of substrate maintained for enzyme production were 0.25%, 0.5%, 0.75%, 1.0%, 1.25%, and 1.5% [55]. The sterilized production media (pH 8) was inoculated with 2 ml of overnight cultured bacteria and incubated at 30 °C using a rotary shaker at 125 rpm for 72 h. After the end of the fermentation time, the enzyme activities were assayed.

### Purification of pectinase by ammonium sulfate precipitation

The crude enzyme was partially purified using ammonium sulfate precipitation methods described by Ramalingam et al., [62]. To avoid the denaturation of enzymes, all purification steps were carried out in a cold environment, utilizing an ice bath and temperatures of 40 °C. About 150 ml of the crude enzyme was precipitated by the addition of four different saturation levels of ammonium sulfate: 30%, 50%, 70%, and 90%. They then dissolved the enzyme proteins that had been frozen in saltwater and dialyzed them with dialysis membranes after the above steps were done [61].

### Application of pectinases in fruit juice clarification

The purified pectinase was applied in the fruit juice-making process to test the clarity of the juice. Three different fruits which have the signs of physical damage (lemon, mango, and apple fruits) were bought from a fruit market (Akaki Kality, Addis Ababa) and brought to microbiology laboratories for juice preparation [61].

### Juice preparation

Lemon, mango, and apple fruits were washed carefully and chopped into smaller sizes on the side with a sharp knife. Twenty grams (20 g) of each chopped fruit were weighed into separate beakers. Those chopped were treated with crude pectinase, purified pectinase, and untreated samples were kept as controls in which the enzyme was replaced by distilled water. The controls and enzyme-treated samples were incubated in a water bath at 40 °C for 1 h, and the activity was stopped by cooling in an ice bath. After that, each juice was filtered through filter paper before the volume of juice production was measured. After enzymatic treatments, the filtered fruit juice was pasteurized at 60 °C for 20 min. To figure out how much juice was made from each piece of fruit, Rai et al., [20] used the method. The formula is:

3 $$\mathrm{Juice}\;\mathrm{yield}\;\left[\%\right]=\frac{weight\;of\;juice}{weight\;of\;fruits}\ast100$$

### Clarification of juice

Using a UV spectrophotometer and the Shet et al., [26] procedure, the clarity of each fruit juice was measured in terms of percentages of transmittances. Around 8 ml of each fruit juice was taken and chilled in a water bath before adding the pectinase enzyme product, after heating at 40 °C to inactivate any natural fruit enzymes or bacteria present. The enzymes (2 mL) were added to 8 mL of fruit juice. After a 4-h incubation period, the samples were heated for 3 min at 40 °C. The juice was centrifuged for 20 min at 3000 rpm, the supernatant was filtered out with filter paper, and the clarity of the juice was calculated by measuring the absorbance at 660 nm with a UV spectrophotometer. Distilled water was used as a blank, and the clarity was expressed in percentages [28].

### Untreated and treated juice properties

#### PH and acidity

The total titrable acidity of the juices was assessed by titration of the juice sample with 0.2 N sodium hydroxide and the pH of cleared and un-clarified fruits (Lemon, Mango, and Apple) juice samples were measured using a pH meter. The results were presented using the phenolphthalein reagent as an indicator and based on g citric acid per 50 ml of each fruit juice [46].

#### Total soluble solid and reducing sugar content

The total soluble solids (SST) of each fruit (Lemon, Mango, and apple) juice were recorded using a refractometer, and the reducing sugar content of each fruit juices sample was measured by the DNS method [63].

### Viscosity

The viscosity of Lemon, Mango, and apple fruits juice samples was evaluated by viscometer at a share rate of 75 rpm and room temperature.

### Pectin presence test

The pectin test was performed on Lemon, Mango, and Apple fruit juice samples by combining cold ethanol with each sample and storing it in the refrigerator overnight. The presence of supernatant indicates the presence of pectin in the sample, while the absence of supernatant suggests the absence of pectin [53].

### Total phenol content

The Folin-Ciocalteu technique was used to determine each fruit juice sample [23, 25]. The total phenol content of the sample was determined by comparing it to the Gallic acid standard curve, with the result represented in mg gallic acid equivalent per ml juice sample (mg GAE/ml)[49].

### Determination of antioxidant activity

The ABTS and DPPH radical scavenging activities of each fruit juice sample were determined using a modified version of the method published by Hosseini et al., [53]. An ABTS solution (6.5 mM) was combined with a potassium persulfate solution (3.5 mM) and stored in darkness for 18 h to make an ABTS solution. After vortexing, the obtained mixtures were kept in darkness for 45 min to complete the antiradical reaction. 0.2 ml of each fruit juice sample was diluted 50 times with distilled water and added to 1.8 ml of the obtained ABTS solution and DPPH ethanolic solution (0.1 mM). After recording the absorbance at 734 nm for ABTS radical scavenging activity (ABTS-RSA) and 517 mn for DPPH radical scavenging activity (DPPH-RSA), these antiradical activities were calculated as follows:

4 $$ABTS-RSA\;or\;DPPH-RSA\mathrm{\%}=\frac{Aconrol-Asample}{Acontrol}*100$$

### Statistical analysis

Statistical analyses of data were conducted using IBM SPSS statistics 20 and origin 2019. All tests were performed in triplicates, and data were expressed as Mean ± standard deviation.

## Data Availability

The datasets used and/or analyzed during the current study are available from the corresponding author on reasonable request. The genome sequence data for R5 and W3is available in the GenBank repository under the project of Addis Ababa Science and Technology University (Website: https://www.ncbi.nlm.nih.gov/nuccore/MN932109.1 and https://www.ncbi.nlm.nih.gov/nuccore/1796387721).

## Abbreviations

C: Celsius
g: Gram
ml: Milliliter
mg: Milligram
rpm: Revolutions per minute
μmol: Micromoles
μL: Microliters
nm: Nanomolar
min: Minutes
U: Units
PCR: Polymerase chain reaction
BLAST: Basic Local Alignment Search Tool
DNA: Deoxyribonucleic Acid
